# Human BK Polyomavirus—The Potential for Head and Neck Malignancy and Disease

**DOI:** 10.3390/cancers7030835

**Published:** 2015-07-08

**Authors:** Raquel Burger-Calderon, Jennifer Webster-Cyriaque

**Affiliations:** 1 Microbiology and Immunology Department, School of Medicine, University of North Carolina at Chapel Hill, Chapel Hill, NC 27599, USA; E-Mail: rbc@unc.edu; 2 Department of Dental Ecology, School of Dentistry, University of North Carolina at Chapel Hill, Chapel Hill, NC 27599, USA; 3 Lineberger Comprehensive Cancer Center, School of Medicine, University of North Carolina at Chapel Hill, Chapel Hill, NC 27599, USA

**Keywords:** polyomavirus, cancer, BK polyomavirus, HIV-associated salivary gland disease

## Abstract

Members of the human Polyomaviridae family are ubiquitous and pathogenic among immune-compromised individuals. While only Merkel cell polyomavirus (MCPyV) has conclusively been linked to human cancer, all members of the polyomavirus (PyV) family encode the oncoprotein T antigen and may be potentially carcinogenic. Studies focusing on PyV pathogenesis in humans have become more abundant as the number of PyV family members and the list of associated diseases has expanded. BK polyomavirus (BKPyV) in particular has emerged as a new opportunistic pathogen among HIV positive individuals, carrying harmful implications. Increasing evidence links BKPyV to HIV-associated salivary gland disease (HIVSGD). HIVSGD is associated with elevated risk of lymphoma formation and its prevalence has increased among HIV/AIDS patients. Determining the relationship between BKPyV, disease and tumorigenesis among immunosuppressed individuals is necessary and will allow for expanding effective anti-viral treatment and prevention options in the future.

## 1. Polyomavirus Family

**Figure 1:**
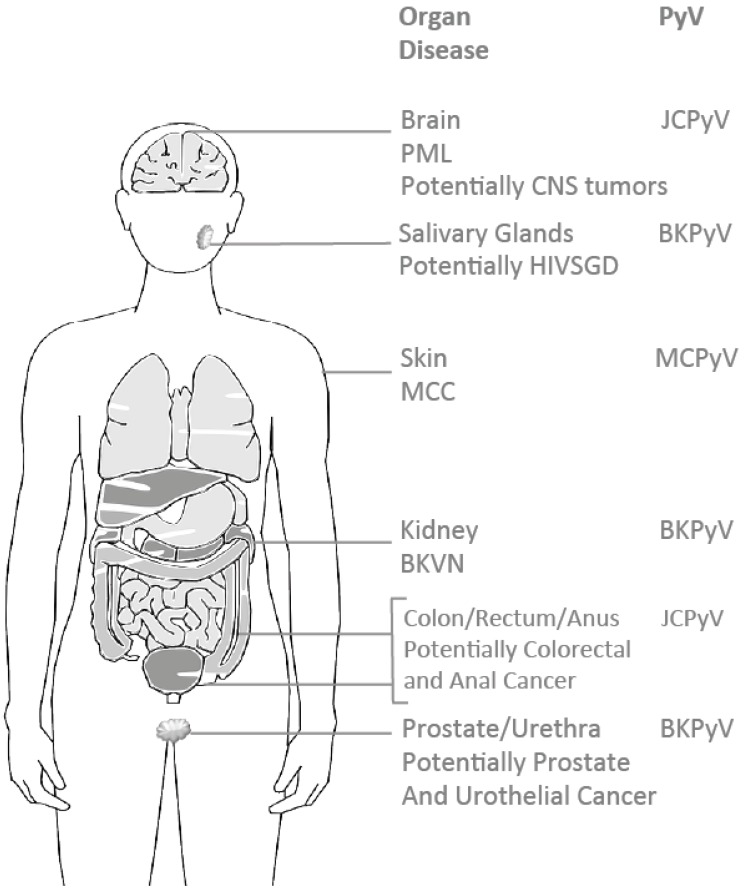
Graphic representation of the human compartments and organs affected by BKPyV, JCPyV, and MCPyV.

The term polyomavirus (PyV) stems from poly- (Greek; multiple) and -oma (Greek; tumors) and was coined when the murine PyV (MPyV) was discovered in neonatal mice with multiple tumors in 1953 [1]. Human polyomaviruses (HPyV) are ubiquitous, opportunistic in nature, and are a rapidly expanding viral cluster. Currently, there are 13 PyV members that infect humans (Table 1). BK polyomavirus (BKPyV) [2], JC polyomavirus (JCPyV) [3] and the more recently discovered Merkel cell polyomavirus (MCPyV) [4] are widely recognized etiological agents of BKPyV-associated nephropathy (BKVN), progressive multifocal leukoencephalopathy (PML) and Merkel cell carcinoma (MCC), respectively [5,6] (Figure 1). The HPyV species KI (KIPyV) [7] and WU (WUPyV) [8] were isolated from the human respiratory tract and characterized in 2007. Trichodysplasia spinulosa-associated polyomavirus (TSPyV) was discovered in skin lesions of immunosuppressed patients with trichodysplasia spinulosa in 2010, and TSPyV is the etiological agent of trichodysplasia spinulosa [9]. HPyV6 [10], HPyV7 [10], HPyV9 [11], and HPyV10 [12,13] were discovered between 2010 and 2012 and are currently not associated with any disease [14], despite HPyV7 recently being detected in human thymoma samples [15]. HPyV10, MWPyV, and MXPyV are the same species but were isolated under different circumstances and time periods [14]. Wieland *et al.* evaluated cutaneous PyV DNA prevalence and viral loads of HPyV6, HPyV7, HPyV9, TSPyV, and HPyV10 by real-time PCR in HIV positive men compared to HIV negative male controls. While HPyV6, HPyV7, TSPyV, and HPyV10 were detected more frequently among HIV positive men compared to HIV negative men (*p* < 0.05) none of the viruses were linked to a particular disease state [16]. St. Louis polyomavirus (STLPyV) was initially isolated from the fecal microbiota of a child in Malawi in 2013 and has since been detected in fecal stool samples from specimens in the US and the Gambia [17,18]. HPyV12, discovered in 2013, was PCR-amplified from liver tissue, as well as from colon, rectum, and feces samples [19]. The New Jersey polyomavirus (NJPyV) is the most recently discovered HPyV and was discovered in a muscle specimen from a pancreatic transplant patient with concurrent retinal blindness and vasculitic myopathy [20].

**Table 1:** List of currently known human polyomaviruses along with respective isolation sources and year of discovery.

HPyV	Year	Source	Reference
BKPyV	1971	Urine, transplant patient	[2]
JCPyV	1971	Brain specimen, Hodgkin’s disease patient	[3]
KIPyV	2007	Nasopharyngeal aspirate	[7]
WUPyV	2007	Nasopharyngeal aspirate	[8]
MCPyV	2008	Merkel cell carcinoma	[4]
HPyV6	2010	Skin swab	[10]
HPyV7	2010	Skin swab	[10]
TSPyV	2010	Nose spicules, trichodysplasia spinulosa patient	[9]
HPyV9	2011	Serum, kidney transplant patient	[11]
HPyV10	2012	Stool sample, child	[12,13]
STLPyV	2013	Stool sample, child	[17]
HPyV12	2013	Liver tissue	[19]
NJPyV	2014	Muscle specimen, pancreatic transplant patient	[20]

Based on epidemiological and phylogenetic data, it has been hypothesized that HPyV co-evolved with their hosts, leading to high prevalence, low pathogenicity and symptom-less latency in immune competent settings [21]. HPyV tend to become latent post-primary infection and undergo reactivation among immune-compromised individuals, including HIV positive individuals, organ transplant patients, and individuals affected by autoimmune diseases [6,22,23,24,25]. Immunosuppressant conditions including pregnancy and chronic alcohol abuse are sufficient to lead to PyV reactivation [6]. The specific factors leading to reactivation are yet to be defined and it has not been determined whether the viruses undergo true latency, expressing only a subset of specific viral genes. Furthermore, it is unclear whether polyomavirus DNA is commonly integrated into the host genome or whether integration is a rare event specific to HPyV subtypes. MCPyV genome integration, for example, is associated with cancer progression [1,4]. The identification of factors associated with reactivation, latency and integration is of increasing importance as the prevalence of human polyomavirus family members and their associated diseases are increasing rapidly.

All HPyV members are small non-enveloped viruses with a ~45 nm diameter, contain a single circular double-stranded DNA genome of ~5.2 kb, and express at least five viral proteins [18]. While the molecular makeup has only been sequence-deduced for the newly discovered HPyV, the genomes contain two distinct transcriptional units: The early region, encoding the alternatively spliced large T antigen (Tag) and small tag; and the late region, encoding the structural viral proteins VP1, VP2, and VP3, that form the viral capsid [18]. The early and the late fragments are separated by a non-coding control region (NCCR) containing a bi-directional promoter and the origin of viral DNA replication [18]. JCPyV, BKPyV, and SV40 (as well as other monkey polyomaviruses) encode for a sixth protein: the agnoprotein [18]. The agnoprotein is a small polypeptide thought to have numerous functions, including the regulation of viral gene expression [26]. Most importantly, all HPyV contain a potentially oncogenic Tag variant [18]. While none of the HPyV seem to encode a middle Tag, which is present in MPyV, alternatively spliced variants of large Tag have been characterized for different HPyV families [18]. For example, MCPyV encodes a 57k Tag [27,28] and JCPyV encodes T’135, T’136, and T’165 [29].

## 2. The Oncogenic Potential of Human Polyomaviruses

The best-studied member of the PyV family, while not considered to be a human pathogen, is the simian vacuolating virus 40 (SV40) [30]. Discovered in 1960, SV40 was one of the first DNA tumor viruses to be described, and its discovery was closely tied to the polio vaccine development in the 1950s. Polio vaccines, along with other viral vaccines, were prepared in primary cell cultures of rhesus monkey kidney cells, some of which contained SV40 [31]. Soon after, SV40 was revealed to induce salivary gland carcinomas in murine neonates [32] and mesotheliomas, lymphomas, brain tumors, bone tumors, and sarcomas in hamsters [33]. In human biopsies, tumors equivalent to the hamster types have been found to contain SV40 DNA and proteins, which is why mesotheliomas and brain tumors have been most consistently linked with SV40 [33]. However, the range of SV40 disease associations has varied from 0 to 100% [33]. While SV40 may be a co-factor in human carcinogenesis, it appears unlikely that SV40 infection alone is sufficient to cause human malignancy as definite links to cancer-formation have not been detected in humans and no cancer epidemics were observed following the administration of polio and adenovirus vaccines contaminated with SV40 [1,29].

SV40 underwent intensive investigation immediately after the potential public health risk posed by the distribution of SV40 contaminated vaccines [31] and the subsequent findings became central to both molecular and cancer biology [34,35]. Thanks to SV40, transcriptional regulation enhancers, alternative splicing, eukaryotic chromosomal DNA replication characterization, identification of tumor suppressor protein p53, elucidation of viral effects on cell cycle regulation, and identification of a protein nuclear localization signal was achieved [31]. Moreover, the extensive studies focusing on SV40 identified the two major viral oncoproteins, large Tag (90–100 kDa nuclear protein) and small tag (17–22 kDa), which are present in all human polyomaviruses (HPyV) [34]. SV40 achieves cellular transformation by using Tag to bypass vital cellular checkpoints. The host cell is forced into the cell cycle S phase, bypassing apoptosis by inactivating the tumor suppressor p53 via Tag binding (inactivating functional activity of p53) [34,35]. RB and other small pocket protein family members, p107 and p130, also bind to large Tag that prevents interaction with E2F1 (a transcription factor responsible for controlled expression of cell cycle-promoting gene), causing the loss of suppression [34,36]. Thus, Tag binding p53 and pRb leads to highly proliferative and uncontrolled cell growth. Studies have shown that Tag interacts with other host proteins, including hsc70, CBP/p300, Cul7 IRS1, Fbxw7, Bub1 and transcription factors including AP-1, AP-2, Sp1, TEF-1, TBP, TAF1 (TAFII250), TAF4 (TAFII135), and TAF5 (TAFII100) [34,37]. Small tag is considered to be an oncogenic enhancer and interacts with the tumor suppressor serine-threonine protein phosphatase 2A (PP2A), which mediates transformation-enhancing signaling pathways [34,37]. Together Tag and tag complete the tumorigenic potential for SV40 and potentially for all HPyVs [34,37].

The oncoprotein Tag is thought to drive transformation of BKPyV laboratory infection in rodents (neonate hamsters, rats and mice) and *in vitro*, inducing tumorigenesis or transformation [38,39,40]. Furthermore, transgenic models established the oncogenic role of MCPyV and JCPyV Tag and tag [41,42,43]. Transgenic mice encoding for MCPyV tag displayed the robust malignant transformation ability of tag, as tag-expressing embryos exhibited hyperplasia, impaired differentiation, increased proliferation, apoptosis, and the activation of a DNA damage response [41]. Interestingly, the epithelial transformation process did not depend on the tag-PP2A interaction, but was strictly linked to a recently described Fbxw7 domain [41]. Another murine model, expressing small and large MCPyV T antigens, displayed hyperplasia, hyperkeratosis, and acanthosis of the skin with additional abnormalities in whisker pads, footpads, and eyes [42]. Additionally, neoplastic progression within stratified epithelia was evident with increased cellular proliferation, unscheduled DNA synthesis, increased E2F-responsive gene levels, disrupted differentiation, and the presence of a DNA damage response [42]. As for JCPyV, infection of primary hamster brain cells generated rapidly growing cells that exhibited numerous characteristics of a transformed phenotype, such as growth in low serum, enhanced production of plasminogen activator, and anchorage-independent growth [43,44,45]. JCPyV infection in Syrian hamster brains *in vivo* led to tumors that included medulloblastomas, primitive neuroectodermal tumors, astrocytomas, glioblastoma multiforme, and peripheral neuroblastomas [43,46,47,48].

The “hit and run hypothesis” is a mechanism deemed valid to justify a co-factorial role of HPyV (other than MCPyV) in cancer onset and progression in humans [31,49]. The hypothesis suggests HPyV causing the host cell cycle to reach the critical point of no return during oncogenic transformation without completing the full viral life cycle [49]. Hence, Tag gene expression, leading to inactivation of p53, without evidence of a productive infection (*i.e.*, viral protein expression, genome replication, *etc.*), leads to host cell transformation [49]. Causality based on this theory is hard to prove experimentally since the complete disappearance of the virus in tumor cells is likely, as HPyV Tag paves the way for tumorigenic transformation without the host cell actually supporting a full viral life cycle [49].

A possible auxiliary role of HPyV in cancer was suggested based on the following premises: (i) Viral oncoproteins transactivate promoters of other oncoviruses present in the host cell, upregulating the oncogene expression of the superinfecting virus, which in turn would enhance the neoplastic development of the host cell. (ii) Oncoviruses are known to evade the innate immune system efficiently, such as by down-regulating TLR expression, and could enhance the likelihood of a superinfection with other oncoviruses [14]. Hence, HPyV may synergize with other viruses in oncogenic transformation [14].

Furthermore, PyV infection poses a unique genotoxic threat to the host cell by inducing a DNA damage response [50]. DNA damage induces a potent cellular DNA damage response (DDR) to maintain genomic integrity [50]. The two protein kinases, ATM (ataxia telangiectasia mutated) and ATR (ataxia telangiectasia and Rad3 related), are major regulators of DNA damage recognition and repair and recent investigation proposes that these essential DDR proteins are required for productive PyV infection [50]. The induced DNA damage responses on the other hand may affect host genomic stability and be an additional oncogenic driver [50].

MCPyV, isolated from Merkel cell carcinoma (MCC) in 2008 [4], is the only recognized HPyV to cause cancer [1,4,51]. MCPyV, a 2A carcinogen (probably carcinogenic to humans), is the etiological agent of MCC [51]. MCC incidence increases with increased age, immunodeficiency, and sun exposure [51,52]. MCPyV is detected in the majority of analyzed MCC biopsies [1,51], but MCC tumor cells do not produce detectable viral particles [53,54] consistent with integration of the viral genome. PCR-amplified MCPyV DNA has frequently been reported among non-melanoma skin cancers of immunosuppressed patients, such as squamous cell carcinoma, basal cell carcinoma and Bowens Disease [1,55]. The specific link between MCPyV and MCC was solidified by studies published between 2008 and 2012. Initially, MCPyV DNA presence was investigated in 5 MCC patients and 10 small cell lung carcinoma (SCLC) patients (both being small neuroendocrine carcinomas), where 40% of MCC specimens but no SCLC specimens were MCPyV positive [1,56]. MCPyV DNA presence was subsequently investigated in 25 pediatric brain tumors, 30 lung tumors, 28 prostate tumors [36,57], and dried blood spot samples of children who later developed leukemia [1,58]. None of these samples were MCPyV DNA positive. Finally, in a more extensive study of 1241 tumors, 10 MCC biopsies that were PCR-amplified were found to be MCPyV DNA positive, but none of the other cancers analyzed (melanoma, basal cell carcinoma, uterine cervix, uterine cervix, large bowel, ovary, breast, bone, and soft tissue) were positive for MCPyV DNA [59]. Thus, MCPyV was confirmed as the etiologic agent of MCC but of no other cancer.

MCPyV genomes are clonally integrated in approximately 85% of all MCC cases and all recuperated integrated genomes and MCC cell lines carry the same signature mutations in the early gene transcripts encoding for Tag [28]. These mutations are important because they selectively abolish viral replication but maintain the oncogene Rb-binding ability, suggesting that the Rb fragment found within the viral protein Tag plays a crucial role during MCC pathogenesis [28]. Similar mutations that potentially lead to oncogenesis may be identified among other HPyVs in the future. While *in vitro* systems allow the study of viral replication post-transfection [28], MCPyV serial transmission has not been possible. The specific human cell type or tissue supporting MCPyV virion production has not yet been identified. However, UV irradiation induces increased MCPyV small Tag transcription *in vivo*, which may rationalize the association between sun exposure, MCPyV infection, and MCC incidence [51,60].

Interestingly, a study testing the presence of MCPyV Tag in 58 samples from diverse CNS malignancies by quantitative real-time PCR found 34 (58.6%) positives, of which 19.0% were schwannomas, 13.8% were meningiomas and 5.2% were pituitary adenomas [61]. While the difference between MCPyV positivity in different types of CNS malignancies was not statistically significant (*p* =  0.066), a multiple linear regression analysis revealed statistically significant differences in MCPyV copy number between meningioma and other CNS tumor types, when the model was adjusted for age and sex (*p* =  0.024) [61]. Hence, the study adds evidence of MCPyV Tag sequence detection among human CNS tumors, despite the numbers being low.

Among the known HPyV members (other than MCPyV), only BKPyV and JCPyV are accredited to be likely to cause human cancers and are classified as 2B carcinogens (possibly carcinogenic to humans) (Figure 1). Clearly, extensive research is required to elucidate the molecular mechanisms underlying viral oncogenic activity and confirm a virus as the etiological agent [49]. While none of the recent studies conclusively corroborate this association (except for MCPyV), evidence continues to accumulate. For example, the JCPyV, which linked to leukoencephalopathy (PML) among patients with suppressed immune systems (most of whom have either hematologic malignancies (13%) [62], receive immunosuppressive therapy (7%) [63] or are diagnosed with AIDS (80%) [29,62,64,65]) is possibly involved in the pathogenesis of colorectal cancer (CRC) [65,66]. CRC is the third most common cancer in females and the fourth most common cancer in males and caused over 694,000 deaths in 2012 alone [67]. JCPyV infects and transforms *in vitro* cultures and induced tumors in laboratory animal models [66,68]. Data suggests that in an infected human colon, partial JCPyV DNA integration, along with additional events may lead to tumorigenesis and cancer progression [66]. The oncogenic potential of JCPyV may be mediated by the PyV oncogene Tag and tissue-specific JCPyV tropism. Tissues supporting permissive JCPyV replication may be lysed, such as oligodendrocytes, whereas cells lacking this tolerance (for example colorectal epithelial cells) may undergo malignant cell transformation [65,69]. Interestingly, JCPyV DNA has been detected in various neoplastic lesions such as oligodendroglioma, astrocytoma medulloblastoma, ependymoma, glioblastoma as well as colorectal carcinoma, gastrointestinal and anal cancers [29,65] (Figure 1), despite the lack of confirmed JCPyV-mediated etiology.

Rennspiess *et al.* recently detected HPyV7-specific nuclear hybridization signals within the neoplastic epithelial cells of 23 thymomas (62.2%) [15]. Of the 20 hyperplastic thymi analyzed, 40% were HPyV7-positive by PCR and confirmed by FISH and IHC in the follicular lymphocytes, whereas all 20 fetal thymi tested HPyV7-negative [15]. The presence of HPyV7-DNA and large Tag expression in the majority of thymomas may link HPyV7 to humanthymomagenesis [15]. However, further investigations are needed to confirm the association between HPyV7 and human thymomas.

A causal relationship was proposed between BKPyV reactivation and the development of bladder cancer in renal transplant patients based on elevated urine BKPyV VLs and the presence of BKPyV DNA in bladder cancer biopsies [70]. The study described a patient that had persistent elevated BKPyV viruria after renal transplantation with subsequent bladder cancer formation (13 months post-kidney transplantation) and dramatic urine BKPyV VL drop after removal of the bladder cancer [70]. Plus, BKPyV DNA was found in the marginal and central part of the bladder tumor [70].

Likewise, there is data suggesting BKPyV to be the etiological agent of prostate cancer. Prostate cancer is a common tumor in Western countries and abrogated p53 function is thought to contribute to prostate cancer risk [71]. BKPyV may contribute to prostate cancer development by Tag-mediated p53 interactions [71]. Indeed a quantitative PCR-based study searching for BKPyV viral DNA among clinical prostate cancer samples found that viral DNA copy numbers were higher in cancer tissues taken from higher Gleason score patients as compared to patients with lower Gleason scores [71]. Furthermore, different p53 mutations were found according to tumor-advanced stages and a statistical significant correlation was found between Gleason score and p53 mutational rate [71]. Most recently, in the cancer genome atlas study TCGA, abundant BKPyV was detected in a bladder cancer with associated high levels of Tag expression [72].

Das *et al.* detected BKPyV DNA in the epithelial cells of benign and proliferative inflammatory atrophy ducts of cancerous prostate specimens [73]. Another study from the same group showed that BKPyV was present at a much lower frequency in noncancerous prostates [74]. In healthy prostates, Tag expression was observed only in specimens harboring proliferative inflammatory atrophy and prostatic intraepithelial neoplasia [74]. The study further showed that the p53 gene from atrophic cells expressing Tag were wild type, whereas tumor cells expressing detectable nuclear p53 contain a mix of wild-type and mutant p53 genes, suggesting that Tag may inactivate p53 in the atrophic cells [74].

Several studies have suggested BKPyV to be oral-tropic [75,76,77,78] and recently the question whether BKPyV and JCPyV play a role in oral squamous cell carcinoma was posed [79]. Head and neck cancers are the most common cancers worldwide and is estimated that approximately 90% of all head and neck cancers are squamous cell carcinomas [79]. Infectious agents, such as viruses are one of many risk factors [79]. Polz *et al.* analyzed the correlation between BKPyV infection and oral squamous cell carcinomas, while accounting for alcohol abuse, tobacco smoking, demographic data, pre-treatment staging, metastases of lymph node evidence, and grading [79]. The study included 92 patients diagnosed with oral squamous cell carcinoma and the results determined that BKPyV DNA was statistically more frequently detected among patients with squamous carcinoma as compared to the control group (*p* < 0.05) [79]. Thus, BKPyV DNA was detected in 18.5% of patients with oral squamous cell carcinoma but only in 3.3% of the controls [79]. JCPyV DNA has been detected in human tonsil tissue, providing evidence as site of infection [80,81] but there is no evidence connecting JCPyV to squamous cell carcinomas [79]. Importantly, a study evaluating the presence of SV40, BKPyV and JCPyV DNA among oral squamous cell carcinoma specimens did not find a significant difference between the cases and the controls, contraindicating a major role of any of these PyV in the etiology of oral squamous cell carcinoma [82].

## 3. BK Polyomavirus (BKPyV)

BKPyV was initially isolated from the urine of a renal transplant patient in 1971 [2] and named after the patient’s initials. BKPyV, as most members of the HPyV family, is holoendemic and found at high frequencies throughout most human populations [21]. BKPyV transmission is still undefined but may include the following courses: respiratory route during infancy [83] fecal-oral route [21], urino-oral route [6], or via vertical transmission [84]. While primary BKPyV infection commonly occurs without symptoms, in rare cases it can cause clinical complications such as urinary tract disease [85]. Upon reactivation BKPyV is mainly associated with three major clinical syndromes: ureteral stenosis, hemorrhagic cystitis and BKPyV-associated nephropathy (BKVN) [86].

**Figure 2:**
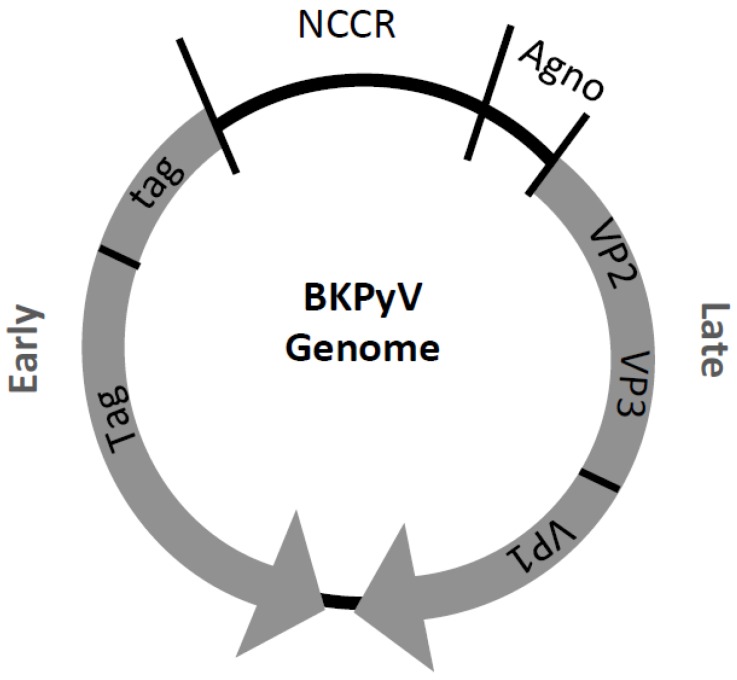
Graphic representation of the BKPyV genome.

BKPyV consists of a naked icosahedral virion with a diameter of about 45 nm and is morphologically indistinguishable from other HPyV [29,87]. The capsid is constituted of 72 capsomers, made of the major structural protein VP1 and one copy of a minor structural protein VP2 or VP3, which also links the genome to the capsid structure [88]. The viral genome is a circular, double-stranded ~5kb DNA molecule and is divided into three functional regions (Figure 2): the early region; the late region; and the promoter, termed NCCR. The BKPyV NCCR promoter region is of bidirectional nature, consists of five block sequences, controls transcription of the early and late genes and has been found to be the major determinant of *in vitro* replication [89,90]. The O block contains 142 bp, the P block 68 bp, the Q block 39 bp, the R block 63 bp and the S block 63 bp. The O block contains the origin of DNA replication and each block carries a combination of transcription factor binding sites (TFBS) [78,87]. TFBS AP-1, CREB, NF-1, C/EBP, NFAT, NFκB, p53, Sp1, and Tag, among others, have been described within the BKPyV NCCR [90,91,92,93,94,95,96]. The early region encodes the Tag regulatory proteins: large Tag (80.5 kDa), small tag (20.5 kDa) and the truncated Tag (17 kDa), which arise from three different mRNAs by alternative splicing of a single primary transcript [88].

Polyomaviruses in general depend on the host cell for replication, as they do not encode for their own polymerase. As discussed above, BKPyV Tag in particular carries numerous and indispensible functions and displays 76% sequence similarity to the well-studied SV40 Tag [97]. Tag acts as major regulatory protein and interacts with pRb and p53 through its domains in order to overcome the host cell cycle control and disrupt host apoptosis (see above for more details). Furthermore, Tag carries DNA unwinding/helicase activity and regulates viral DNA replication and gene expression by interacting with host-cell transcription factors and the viral promoter region (NCCR) [21]. The late region encodes the three structural proteins VP1 (40.1 kDa), VP2 (38.3 kDa), and VP3 (26.7 kDa), and the non-structural agnoprotein (7.4 kDa). Proteins VP2 and VP3 are translated from the same transcript and the agnoprotein and protein VP1 are translated from a different open reading frame (ORF) [88]. The agnoprotein is a small, non-immunogenic lipid-associated cytoplasmic protein with an elusive functional nature even though it has been thought to be important for gene expression, capsid assembly and egress [26]. The late BKPyV genome region contains additional ORFs, which are not well studied, but may encode a protein similar to the SV40 VP4 protein [98].

## 4. BKPyV-Associated Renal Diseases

BKPyV has been studied mainly due to the severe complications reactivation causes among transplant patient even though symptomatic BKPyV infection is also known to cause interstitial inflammations, renal tubular atrophy, pneumonia and meningoencephalitis in other settings [99,100]. En estimate of ten percent of renal transplant patients with increased BKPyV viremia and viruria will develop BKVN (Figure 1), which leads to graft loss in 90% of the cases [101]. BKVN has developed into a new epidemic, becoming the most important infectious complication affecting kidney transplants over the past eight years. The worldwide BKVN incidence rate lies within 1% and 9% (6.5% at the University of North Carolina at Chapel Hill, Chapel Hill, NC, USA) among children and adults and has been increasing due to the development of potent immunosuppressive drugs [102].

## 5. HIV-Associated Salivary Gland Disease (HIVSGD)

HIV-associated salivary gland disease (HIVSGD) is currently the most common salivary gland presentation in HIV positive individuals [103]. Incidence is as high as 48% among HIV-infected patients in developing countries [104] and it is more commonly diagnosed among the pediatric population, while being considered AIDS-defining among children. Importantly, HIVSGD is considered a pre-malignant lesion and its diagnosis is associated with increased lymphoma incidence [76,105,106,107]. Schiodt *et al.* define HIVSGD as a disease which encompasses symptoms linked to AIDS-related salivary lymphadenopathy such as enlargement of the major salivary glands and/or xerostomia (dry mouth) and localized lymphocytic infiltration [108]. HIVSGD presents itself superficially as unilateral or bilateral salivary gland enlargement due to parotitis. Histologically, the oral disease is characterized by hyperplastic, intraparotid lymph nodes and/or lymphatic CD8 + infiltrates [103]. Parotid gland enlargements are greater and more disfiguring in children than in adults [76]. HIVSGD also affects the minor salivary glands, with labial salivary glands demonstrating features of sialadenitis [76]. Patients diagnosed with HIVSGD commonly have reduced salivary flow rates of the parotid, submandibular, and sublingual glands [103]. Saliva composition may be affected and may contain increased sodium, chloride, lysozyme, peroxidase, lactoferrin, and immunoglobulin A levels [103].

Salivary gland disease impacts oral health, and maintenance of good oral health among HIV/AIDS patients is of outmost importance to protect the patients from secondary and opportunistic infections [109]. Bacterial, viral and fungal infections that begin in the mouth may escalate to systemic infections and may in turn harm vital organs if left untreated [109]. Moreover, the decline of oral health has been shown to impact the patient’s quality of life by limiting career opportunities and social contact as a result of facial appearance and odor [109]. Poor oral health may also lead to complications with food intake, leading to general malnutrition and malabsorption of vital medication [109]. Poor oral health further predisposes the development of oral diseases, such as xerostomia. Xerostomia leads to dental decay, periodontal disease and increases the patient’s likelihood of being affected by pathogenic opportunistic infections [109].

**Figure 3:**
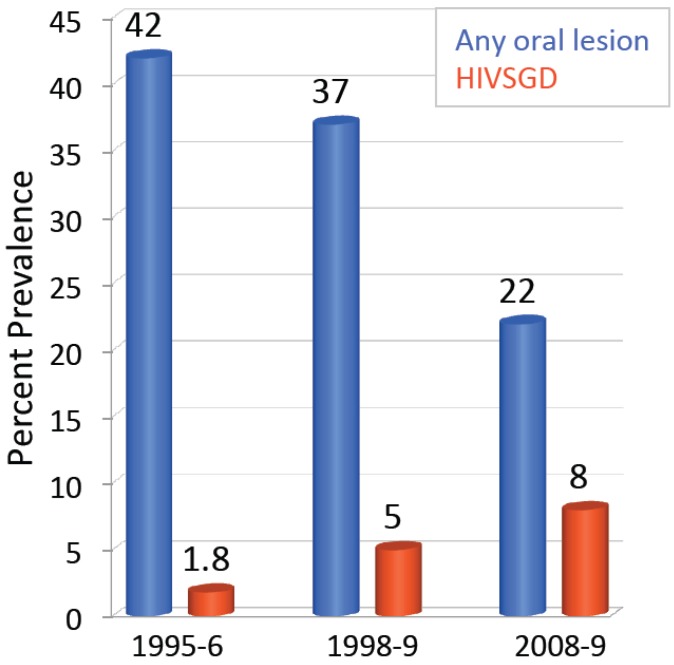
HIVSGD (red) has increased during the HAART era compared to all other HIV-associated oral lesions (blue) at the UNC Hospitals from 1995 to 2009.

Interestingly, there has been an increase in HIVSGD prevalence of among HIV positive patients in the post-highly active antiretroviral therapy (HAART) era at the UNC hospitals (Figure 3) [110,111] and was detected at a rate of 8% in the UNC hospital cohort in 2010. Taking a combination of three or more anti-retroviral drugs is termed HAART and its application dramatically increases the life expectancy of HIV positive individuals. HIVSGD has risen from 1.8% to 5% among HIV-infected adults from 1995 to 1999, even though the overall presence of oral lesions has decreased [110]. Patton’s data implies that AIDS patients were experiencing a higher risk of developing HIVSGD under HAART treatment. The group also determined that there was a change in the occurrence of oral opportunistic infections in general from 1995/96, where the use of HIV protease inhibitor was less common, to 1999, a period of greater protease inhibitor use, indicating the importance of understanding opportunistic pathogens [110]. HAART hinders AIDS progression by reducing HIV RNA levels and increasing CD4+ cell counts, but it has been shown that opportunistic infectious agents can also take advantage of the newly reconstituted immune system.

## 6. HIVSGD-Lymphoma Association

HIVSGD associated salivary gland enlargement may represent a consequence of cystic lymphoepithelial lesions [76,112]. These lesions reflect a localized manifestation of persistent generalized lymphadenopathy [112]. While HIVSGD is regarded as a benign condition, malignant lymphoma has been described in association with some of these lesions [112]. Importantly, lymphomas represent a significant proportion of malignancies of the major salivary glands, accounting for 1.7% to 7.7% of tumors [112]. Most HIV-related lymphomas and lymphoma-like lymphoproliferative disorders are aggressive B-cell proliferations, where the incidence of non-Hodgkin lymphoma in HIV positive individuals remains approximately 70 to 80 times greater than that of the general population [113]. Interestingly, the epidemiology of these neoplasms has changed with the introduction of HAART [113]. HIV-associated non-Hodgkin lymphomas now account for most AIDS-defining cancer types, despite the overall incidence having decreased [113]. While the salivary B cell lymphoma is rather indolent and tends to remain localized, as the disease progresses, regional lymph node involvement or other extra-nodal site involvement may take place [114].

Early on, the lymphomas were solely linked to Epstein-Barr virus (EBV). A study observing six cases of primary salivary gland lymphoma in HIV positive patients found that all of them were of high histologic grade while salivary gland lymphomas unrelated to HIV were predominantly of low-grade MALT type [115]. While the lymphomas in both categories expressed the B-cell phenotype EBV RNA transcripts (EBER) were demonstrated in three, and EBV latent membrane protein (LMP) in two of the six HIV-related and in none of the three HIV-unrelated lymphomas [76,115]. However, with new findings connecting the head and neck compartment to BKPyV, one has to ask whether infectious agents other than EBV may play co-factorial roles during oncogenesis.

## 7. BK Polyomavirus HIVSGD Association

Since Jeffers *et al.* detected significantly higher BKPyV viral loads (VLs) in the saliva of patients diagnosed with HIVSGD as compared to HIV negative patients [75], evidence linking BKPyV to HIVSGD has augmented. Jeffers *et al.* showed that the oral BKPyV VLs of HIVSGD patients (*n* = 11) ranged from 10^1^ to 10^4^ copies/mL whereas the HIV negative control cohort (*n* = 7) VLs ranged from 0 to 10^2^ copies/mL. The higher BKPyV levels shed in an HIVSGD cohort were confirmed two years later [76]. The study found highest BKPyV VLs in patients diagnosed with HIVSGD (*n* = 11) as compared to patients who were HIV positive without HIVSGD (*n* = 46) and HIV negative individuals (*n* = 12). Furthermore, using PCR and immunofluorescence, BKPyV products (but not herpesviral DNA) were found in HIVSGD patient salivary gland biopsies but not in biopsies from patients without HIVSGD [116]. Hence, BKPyV sequences were detected among most HIVSGD throatwash (TW) samples and biopsies. Significantly lower BKPyV levels were detected among HIVSGD negative TW samples and no BKPyV sequence were found among biopsies from patients without HIVSGD. Therefore, based on Fredrick’s and Relman’s postulates, which integrate modern sequence-based identification of microbial pathogens, a link between BKPyV and HIVSGD is likely.

BKPyV has been shown to display oral tropism *in vivo* and *in vitro.* BKPyV has been detected in tonsillar tissue from both pediatric and adult donors [83,117,118] and Jeffers *et al.* showed in 2009 that BKPyV is able to infect and reproduce in human salivary gland cells *in vitro* [75]. Laboratory strain BKPyV underwent entry, transcribed, translated and produced virions within human submandibular (HSG) and parotid (HSY) salivary gland cells. It is plausible therefore that the oral compartment represents an infectious reservoir in addition to the historically described renal/urinary compartment [102,119]. Previous studies have shown that the replication compartments of urine and plasma-derived BKPyV are distinct *in vivo* [120,121], similarly, the salivary gland may embody a separate replication compartment in the HIVSGD setting [122]. Finally, clinical HIVSGD-derived BKPyV isolates infect and complete their full viral life cycle and produce infectious viral progeny in human salivary gland cells and provide evidence of preferred oral tropism, as oral-derived BKPyV isolates replicated more efficiently in HSG cells than in kidney cells [78].

Detecting an increase in prevalence of HIVSGD among HIV positive patients in the HAART era may further hint towards persistence of an opportunistic infectious agent as the cause of HIVSGD [123]. Twenty-five to 35% of patients undergoing HAART develop a pathological inflammatory response termed immune reconstitution inflammatory syndrome (IRIS) to previously treated or asymptomatic opportunistic infections. Interestingly, the majority of IRIS cases are reported within the first two months of HAART, even though IRIS development can take up to two years [124].

Antigen-driven HIVSGD pathogenesis provides further evidence of an infectious agent being the etiological agent of HIVSGD. A prospective study analyzing epidemiology, clinical presentation, and extra-glandular manifestations of HIVSGD suggests that this lymphadenopathy is an antigen (viral)-driven response [125]. Additionally, an antigen-driven MHC-determined host immune response recorded by Itescu *et al.*, based on the infiltrating lymphoid cells characterizing HIVSGD, points to an antigen-driven response [126]. Finding differential rates of HIVSGD in children (20%–47%) and adults (3%–7.8%) [107] may also allude to a viral infection as it indicates primary viral infection in children *versus* residual immunity in adults [76]. However, future trials will have to address these questions more specifically.

## 8. Potential Factors Allowing for BKPyV Tropism in the Head and Neck

Members of the polyomavirus family have a narrow species host range but infect a wide range of cell types within a host [86], yet it is not clear which factors determine BKPyV tropism. BKPyV capsid proteins interact directly with the receptor molecules as infection is initiated, since it is a non-enveloped virus. This interaction is generally thought to be the major determinant of viral host and tissue tropism [127]. BKPyV binds to cellular receptors such as N-linked glycoproteins with a 2,3-linked sialic acids and gangliosides GD1b and GT1b. This is true for kidney (Vero) cells and oral (HSG) cells *in vitro* [75]. BKPyV is subsequently internalized via caveolae-mediated endocytosis, and is transported towards the endoplasmic reticulum (ER) via the host cell cytoskeleton. Mutations in the major capsid protein VP1 may lead to differential host cell receptor binding. Neu *et al.* [128] described the structural requirements that underlie receptor switching and showed that the amino acid at position 68 in VP1 is a determinant of receptor specificity. The *in vitro* experiments show that a lysine to serine mutation of this residue switches the receptor specificity of BKPyV from GD3 to GM1. These findings emphasize the plasticity of viral receptor binding sites and potential host cell type retargeting mechanism. During infection, BKPyV undergoes uncoating once at the endoplasmic reticulum (ER), and nuclear localization signals (found on the minor capsid proteins VP2 and/or VP3) further direct viral genomes to the nucleus and are subsequently imported via the host’s nuclear import machinery [88]. Maraldi *et al.* showed that this process is completed within 12 hours [129]. Once in the nucleus, the early genes are expressed and the BKPyV genome is replicated before late gene expression starts and virions are assembled in the nucleus [21]. Given that receptor interactions may not be the only factors determining tropism, JCPyV tropism may be determined by its promoter [90,127]. It is important to consider the contribution of transactivating factors (regulating BKPyV gene expression) to tropism [90,127]. There is evidence that downstream events, such as endocytosis, virus-induced signaling, intracellular trafficking and transcriptional regulation contributes significantly to viral tropism [127].

As mentioned earlier, the BKPyV NCCR promoter contains the origin of replication and the enhancer/promoter elements of the genome and is the main determinant of BKPyV replication *in vitro* [89]. The NCCR is a hypervariable region and comparative studies have suggested that it may regulate host cell tropism mainly due to the rearrangement, duplication or deletion of TFBS [90,127,130]. It is therefore plausible that the interplay of TFBS found within the NCCR sequence of a certain BKPyV substrain and transcription factors present within a certain cell type may allow for successful completion of a viral life cycle and therefore determine BKPyV tropism (similar to JCPyV).

The BKPyV NCCR commonly undergoes block deletions and/or duplications as compared to the archetype. Recently the NCCR architecture from oral-derived HIVSGD BKPyV has been characterized and a unique OPQPQQS BKPyV NCCR promoter sequence detected among two clinical HIVSGD-derived BKPyV isolates [78]. Promoter architectures that diverge from the original urine-derived archetypic OPQRS arrangement are referred to as rearranged (rr) and are readily detected *in vitro* and *in vivo* [131,132,133,134,135,136,137,138,139]. It has been shown that NCCR block rearrangements bestow remarkable differences in transforming potential and host cell permissiveness [90,120,121,132,137,140]. Clinical studies determined that the emergence of rr NCCR BKPyV variants in the plasma samples from immune suppressed kidney transplant recipients were correlated to increased replication efficiencies and pathogenesis [120]. Furthermore, rearranged NCCRs have been reported to be more efficient replicators *in vitro* [89,90,134,141,142].

Similar to BKPyV, the JCPyV promoter has been found to rearrange, and rearrangements of following substrains isolated from brain, kidney, lymphocytes of PML patients, brain, cerebrospinal fluids (CSF), and lymphocytes of healthy individuals have been described [143,144,145,146,147]. JCPyV promoter region includes binding sites for a variety of transcription factors, similar to the BKPyV NCCR. Interestingly, within JCPyV it is thought that these rearrangements lead to alterations of the TFBS and hence transcriptional patterns of the promoter, which are thought to increase the viral replication capacity in the brain and ultimately the viral disease potential [148]. It is important to note that the correlation between promoter structure rearrangement and disease is not perfect. The same JCPyV promoter architectures can be found within patients with extensive disease and patients without noticeable disease [83], while it is known that following TFBS Tst-1, NF-1, Sp1, GBPi, NFκB, YB-1, Pura, and GF-1 determine the tropism of JCPyV to glial cells in the brain [83,149,150,151,152,153,154,155]. While host factors contribute to the viral pathogenic capacity and likely fluctuate between individuals and over time [83] both JCPyV and BKPyV promoter rearrangements are likely to drive viral replication and therefore pathogenicity *in vivo*. Whether BKPyV rr NCCR variants are more efficient at *in vivo* disease development is debated [156,157] but not unlikely since JCPyV promoter rearrangements have also been suggested to drive neural tropism and bestow increased virulence as discussed above [158]. It is important to conclusively determine whether BKPyV promoter rearrangements truly endow higher replication levels and increased virulence in order to understand BKPyV pathogenesis and prevent disease development effectively. This may be facilitated by either using certain BKPyV NCCR architectures as biomarkers or treatment targets. The promoter architecture from following samples has been described: bladder, brain, cerebrospinal fluid, eye, heart, kidney, lung, muscle, nasopharyngeal aspirates, ovarium, monocytes, placenta, prostate, ureter, urine, and sewage [140].

## 9. Conclusions

Approximately 15%–20% of cancers are thought to be caused by infectious agents [159]. Around 12% of cancers are causally linked to Epstein-Barr virus (EBV), hepatitis B virus (HBV), human papillomavirus (HPV), human T-cell lymphotropic virus (HTLV), hepatitis C virus (HCV), Kaposi’s sarcoma herpesvirus (KSHV), and MCPyV [159]. In the past 15 years renewed interest in HPyV and its prevalence and pathology has been sparked as a result of novel HPyV identifications and the accumulation of evidence suggesting that more than just the HPyV MCPyV may cause cancer. New members of the HPyV family have been emerging as novel opportunistic pathogens among HIV/AIDS patients and immunosuppressed individuals, possibly carrying harmful implications [99]. Additionally, members of the HPyV family have been proposed to be co-factors for cancers induced by other oncoviruses [14], yet there is little known about their life cycles and possible malignant transformation potential among humans and effective antiviral treatments are rarely available. For BKPyV in particular, there is no specific antiviral treatment available, despite BKVN often resulting in chronic allograft dysfunction and failure [160]. Especially in the light of the newly discovered connection between BKPyV and HIVSGD, the deleterious implications of BKPyV on public health become substantial [86]. Clearly, further elucidating the pathological process leading to HPyV- and BKPyV-associated disease and potentially oncogenesis is critical, and the constantly improving molecular techniques for viral detection will have to lead the way.
